# Interfacility Transfer for VA-ECMO in Beta Blocker and Calcium Channel Blocker Overdoses: A Report of Two Cases

**DOI:** 10.5811/cpcem.34869

**Published:** 2025-01-12

**Authors:** Richard Fisher, Santiago Batista Minaya, Heather Brunette, Joshua Nogar, Payal Sud

**Affiliations:** * Northwell Health, Department of Emergency Medicine, New Hyde Park, New York; † Northwell Health, Department of Emergency Medicine, Division of Medical Toxicology, New Hyde Park, New York

**Keywords:** case series, VA-ECMO, toxicology, beta blocker, calcium channel blocker

## Abstract

**Introduction:**

Calcium channel blocker (CCB) and beta blocker (BB) overdoses are life-threatening conditions that can lead to vasoplegic and cardiogenic shock. Treatment involves a combination of vasopressors, calcium, glucagon, and/or high-dose insulin euglycemia therapy. The most severe overdoses may require venoarterial extracorporeal membrane oxygenation (VA-ECMO), which often results in interfacility transfers. This report describes two successful VA-ECMO transfers for refractory CCB/BB overdose.

**Case Reports:**

**Case 1**

A 56-year-old male developed severe hypotension after ingesting 40–45 tablets of 10 milligram (mg) amlodipine tablets. After initial treatment approaches were unsuccessful, an early interdisciplinary discussion facilitated timely cannulation at the initial facility and quick transfer for VA-ECMO initiation. The patient was discharged at his neurological baseline after 60 days.

**Case 2**

A 19-year-old female presented to the emergency department after a polypharmacy ingestion including 60 tablets of 20 mg propranolol. An early interdisciplinary discussion between the medical intensive care unit, medical toxicology, and the ECMO team allowed for prompt transfer directly to the receiving hospital catheterization lab for VA-ECMO within three hours of the initial presentation. The patient was discharged to an inpatient psychiatric facility after nine days.

**Conclusion:**

Venoarterial extracorporeal membrane oxygenation for refractory shock due to CCB and BB overdoses can be a life-saving intervention. Interfacility transfer of poisoned patients for VA-ECMO is logistically challenging, which can delay the appropriate care for patients with an otherwise morbid prognosis. A streamlined interfacility transfer protocol with multidisciplinary collaboration can help optimize outcomes.

## INTRODUCTION

Calcium channel blocker (CCB) and beta blocker (BB) poisonings are associated with significant morbidity and mortality. According to the 2022 Annual Report of the National Poison Data System, CCBs and BBs were ranked sixth and seventh, respectively, for all-cause poisoning mortality, with a combined total of 323 deaths.^1^ Even massive overdoses can be asymptomatic early in the course of a poisoning, but the patient can rapidly develop vasoplegia, followed by cardiogenic shock and dysrhythmias. While both CCBs and BBs can cause cardiotoxicity, their mechanism of action differs. Calcium channel blockers can be divided into nondihydropyridines (diltiazem and verapamil) and dihydropyridines (amlodipine, nifedipine, etc). Nondihydyropyridines act on L-type calcium channels reducing both myocardial chronotropic and ionotropic activity. Dihydropyridines preferentially act on the peripheral vasculature and are potent vasodilators at therapeutic concentrations. In large overdoses, specificity is lost and all CCBs can impact both the vasculature and myocardium. Beta blockers exert a negative chronotropic and ionotropic effect but less of a direct impact on the peripheral vasculature compared to CCBs.

The current treatment approach involves a combination of vasopressors/inotropes, calcium, glucagon, and high-dose insulin euglycemia therapy (HIET). In refractory cases, methylene blue, lipid emulsion therapy, and venoarterial extracorporeal membrane oxygenation (VA-ECMO) have also been used. The use of VA-ECMO for cardiogenic shock secondary to drug poisonings has increased over the past decade.^2^ In its 2023 guidelines, the American Heart Association recommends the use of VA-ECMO for cardiogenic shock and cardiac dysrhythmia.^3^ Frequently, the decision to perform VA-ECMO is a last resort but has been shown to improve acidemia, hemodynamics and, in some studies, mortality.^2^,^4^–^7^ However, a major barrier to the initiation of VA-ECMO is the need for an interfacility transfer, which requires the activation of emergency medical services and multiple specialized medical teams. Within an ECMO-capable health system, a protocol for real-time interdisciplinary discussions between medical toxicology, cardiothoracic and medical intensive care units (ICU), emergency departments (ED), and a transfer center has streamlined the process to determine which poisoned patients may benefit from ECMO therapy. We present two cases of severe cardiogenic shock secondary to CCB and BB blocker toxicity that were transferred successfully for VA-ECMO therapy.

## CASE REPORTS

### Case One

A 56-year-old male with a past medical history of hypertension and hyperlipidemia presented to the ED with a syncopal episode approximately 17 hours after ingesting 40–45 tablets of 10 milligram (mg) amlodipine tablets in a suicide attempt. He denied any coingestants. Initial vitals revealed an oral temperature of 36.3° Celsius, blood pressure of 89/45 millimeters of mercury (mm Hg), heart rate of 83 beats per minute (bpm), a respiratory rate of 18 breaths per minute, and an oxygen saturation of 100% on room air. In the ED, the patient’s blood pressure initially improved to 93/64 mm Hg with norepinephrine at 0.17 micrograms/kilogram/minute (mcg/kg/min). However, his blood pressure began to decline over the next several hours prompting aggressive resuscitation with norepinephrine up to 1 mcg/kg/min, vasopressin at 0.04 units/minute, phenylephrine up to 3 mcg/kg/min, epinephrine up to 0.05 mcg/kg/min, and HIET up to 10 units/kg/hour in conjunction with the medical toxicology team. At this time his blood pressure was 84/50 mm Hg. A multidisciplinary conversation between the presenting hospital’s intensivist, the medical toxicology team, the centralized transfer center, the receiving hospital’s intensivist, and the receiving hospital’s ECMO team determined that the patient would benefit from transfer to an ECMO-capable facility in anticipation of further decline. The patient was then cannulated for VA-ECMO at the presenting hospital and subsequently transferred directly to the cardiothoracic ICU at a VA-ECMO-capable site. Upon arrival, VA-ECMO was promptly initiated.

**CPC-EM Capsule**

What do we already know about this clinical entity?

*Venoarterial extracorporeal membrane oxygenation (VA-ECMO) is an effective intervention for refractory beta blocker and calcium channel blocker toxicity*.

What makes this presentation of disease reportable?

*Two patients successfully transferred to an ECMO-capable site highlights the importance of the interfacility transfer process*.

What is the major learning point?

*A multidisciplinary approach facilitates quick and effective transport to an ECMO center*.

How might this improve emergency medicine practice?

*Emergency physicians should be cognizant of ECMO’s role in certain overdoses, and streamlined interfacility transfer protocols could improve patient outcomes*.

Throughout his hospital course, the patient received HIET (up to 16 units/kg/hr) and VA-ECMO for eight days as well as a combination of vasopressor support (epinephrine up to 0.07 mcg/kg/min, norepinephrine up to 2.10 mcg/kg/min, vasopressin at 0.04 units/min, and angiotensin II at 30 nanograms/kg/min) for a total of 12 days. However, at the time of ECMO decannulation, he required only vasopressin at 0.04 units/min and norepinephrine at 0.04 mcg/kg/min. He was successfully extubated after 14 days of mechanical ventilation but required several days of hemodialysis secondary to acute kidney injury. His medical course was complicated by acute acalculous cholecystitis, lower extremity weakness, and tachycardia. In total, the patient was hospitalized for 60 days but had a complete return to baseline neurologic status at discharge.

### Case Two

A 19-year-old female with a history of major depressive disorder presented to the ED 60–90 minutes after ingestion of 60 tablets of 20 mg propranolol, 30 tablets of 50 mg hydroxyzine, and 90 tablets of 60 mg fluoxetine in a suicide attempt. Initial vitals revealed an oral temperature of 36.5°C, blood pressure of 87/57 mm Hg, heart rate of 82 bpm, a respiratory rate of 16 breaths per minute, and an oxygen saturation of 96% on room air. The patient was somnolent and confused and ultimately developed a generalized tonic-clonic seizure that resolved after 4 mg of lorazepam. She was intubated and orogastric lavage was performed, followed by administration of 50 grams (g) of activated charcoal. The patient became bradycardic to 50 bpm and remained hypotensive, with a minimum blood pressure of 67/37 mm Hg. Norepinephrine at 0.05 mcg/kg/min was started as well as HIET at 1 unit/kg/hr in consultation with medical toxicology. She ultimately required norepinephrine up to 5 mcg/kg/min, epinephrine up to 2 mcg/kg/min, phenylephrine up to 6 mcg/kg/min, 5 mg of glucagon, 3 g of calcium gluconate, and HIET at 1 unit/kg/hr.

An early discussion with the medical toxicology team and a VA-ECMO-capable facility took place following HIET initiation, and the patient was transferred from the presenting ED directly to the catheterization lab at the receiving facility for VA-ECMO within three hours of initial presentation. The patient was successfully decannulated after three days on VA-ECMO. She was extubated on hospital day five and transferred to an inpatient psychiatric facility on day nine. Her course was complicated by a left upper extremity deep vein thrombosis, pneumonia, and heart failure with an ejection fraction of 15% that improved to 55–60% at discharge.

## DISCUSSION

The use of ECMO initially gained recognition in the 1980s as a therapy for neonatal respiratory failure and was quickly recognized as an intervention that could be applied beyond the scope of pediatric cardiac surgery.^8^ The role of VA-ECMO in the context of a poisoning or overdose was first described in 1997 in a 16-month-old with quinidine toxicity. The patient developed refractory bradycardia and hypotension, required 11 days of VA-ECMO, and was discharged neurologically intact.^9^ This case suggested that VA-ECMO could be a life-saving intervention in the treatment of drug-induced shock.

Compared to data for cardiac arrest, however, the role of VA-ECMO in drug-induced shock is limited to observational studies and case reports. A review of the National Poison Data System database from 2000–2018 showed that the utilization of VA-ECMO for the treatment of poisonings has increased, but without a clear mortality benefit.^10^ Complications such as bleeding, limb ischemia, and circuit-clotting from thrombi or lipid emulsion are not infrequent.

The role of VA-ECMO in the treatment algorithm of refractory CCB and BB toxicity is of great interest to medical toxicology, poison control centers, and VA-ECMO teams. Our 21-hospital medical toxicology consult service has seen an increasing number of dihydropyridine CCB and BB overdoses of varying severity, morbidity, and mortality. Currently, there are no national treatment guidelines for the timing and utilization of VA-ECMO in the setting of CCB and BB toxicity. One study found that a higher vasoactive-inotropic score (VIS), a scoring system based on the dosages of vasopressors and inotropes used in resuscitation, prior to ECMO, was associated with greater in-hospital mortality in patients with cardiogenic shock.^11^ However, the study included both ischemic and non-ischemic etiologies for cardiogenic shock, did not consider HIET, and could not be extrapolated to vasoplegic shock.

While our approach does not currently include a specific VIS value for ECMO initiation, it can be used as a generalized framework to ensure that patients with severe hemodynamic instability, or the potential to exhibit it, be expeditiously transferred to facilities where a multidisciplinary bedside evaluation can be performed. For example, in Case Two the patient’s VIS score was initially 5 but rapidly increased to 700 over the span of a few hours prior to transport.

The figure below describes a suggested generalized flowsheet for patients who present with a CCB/BB overdose at a hospital without ECMO capabilities. The ED team consults the medical toxicology team early in the patient’s presentation, regardless of the patient’s hemodynamic status. The medical toxicology team will determine whether the patient could benefit from transfer to an ECMO-capable center for either further monitoring or VA-ECMO initiation. The medical toxicology team initiates a multidisciplinary conversation involving the ED team, ECMO team, ICU, and transfer center. Ultimately, the ECMO team will make the final decision for cannulation if no exclusion criteria are met, with considerable input from medical toxicology.

Our centralized transfer center is then able to direct the transfer of the patient to the facility within our system that can most appropriately care for the patient, based on the current resources available. While no specific hemodynamic triggers exist to discuss initiating VA-ECMO, the conversation will typically occur with increasing vasopressor and HIET support. The ability for this process to occur is in part due to our hospital system’s well-established medical toxicology consult service, as well as a VA-ECMO system in place for cardiac arrests.

According to the Extracorporeal Life Support Organization, there are currently 14 ECMO-capable hospitals in the state of New York. In certain states, this number drops to one or even zero.^12^ Resources are often limited at the initial facility, making cannulation prior to transfer a challenge. Together, these factors delay care and increase the risk of complications from ECMO. Nevertheless, interfacility transfer is still shown to be a feasible approach, with no difference in complications if cannulated at the initial facility vs the receiving facility.^13^,^14^ In Case One, the initial hospital had the capability to cannulate before transfer, preventing a delay in care. However, in Case Two, this was not possible, and the patient was quickly transferred directly to the receiving hospital catheterization lab for cannulation and initiation of VA-ECMO. In both scenarios the patients had good neurological outcomes. The hospital system’s pre-existing transfer policy pertaining to the management of shock due to CCB/BB toxicity likely played a role in these results. A VA-ECMO protocol has been described previously in other hospital systems.^15^

## CONCLUSION

These two cases suggest that VA-ECMO therapy can be used in the setting of severe dihydropyridine-induced vasoplegic shock refractory to aggressive vasopressor support and high-dose insulin euglycemia therapy. This report also emphasizes the effectiveness and importance of establishing an interfacility transfer protocol to VA-ECMO-capable centers. Given the inherent logistical constraints with transferring critically ill patients between facilities, it is important to consider an early transfer of a patient to an ECMO-capable center before life-threatening CCB/BB toxicity has begun to manifest. Successful coordination of care for patients with life-threatening overdoses may require a multidisciplinary conversation involving services unfamiliar with one another (toxicology, cardiothoracic surgery, medical ICU, shock teams, etc) and is best achieved with pre-planning for such an event.

## Figures and Tables

**Figure:**
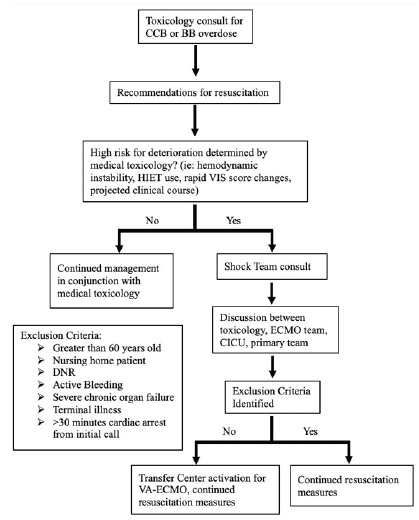
Flowchart for venoarterial extracorporeal membrane oxygenation (VA-ECMO) initiation. *CCB*, calcium channel blocker; *BB*, beta blocker; *HIET*, high-dose insulin euglycemia therapy; *VIS*, vasoactive inotropic score; *CICU*, cardiac intensive care unit; *DNR*, do not resuscitate.
